# Low work ability and high disability burden in Cushing’s syndrome: a multicenter cohort study

**DOI:** 10.1007/s40618-026-02966-5

**Published:** 2026-07-08

**Authors:** Annika Steinkogler, Stephanie Zopp, Kathrin Moser, Júnia R. O. L. Schweizer, Isabel Stüfchen, Friederike Völter, Katrin Ritzel, German Rubinstein, Leah T. Braun, Marily Theodoropoulou, Birgit Linkohr, Rolf Holle, Tina Kienitz, Marcus Quinkler, Martin Reincke, Elisabeth Nowak

**Affiliations:** 1 Department of Medicine IV, LMU University Hospital, LMU Munich, Munich, Germany; 2 Department of Nuclear Medicine, University Hospital of Munich, LMU Munich, Munich, Germany; 3 Institute of Epidemiology, Helmholtz Zentrum München, German Research Center for Environmental Health, Ingolstädter Landstraße, Munich-Neuherberg, Germany; 4 Institute for Medical Information Processing, Biometry and Epidemiology, LMU Munich, Munich, Germany; 5 Endocrinology in Charlottenburg, Berlin, Germany; 6 Ziemssenstrasse 5, 80336 Munich, Germany

**Keywords:** Employment, Social and occupational health, Impairment, Fatigue, Cortisol

## Abstract

**Context:**

The socio-occupational burden of Cushing’s syndrome (CS) remains underrecognised. In clinical practice, surgically treated patients are often considered recovered, potentially overlooking persistent impairment.

**Purpose:**

To evaluate work ability, social and employment status in newly diagnosed and remitted CS.

**Methods:**

National observational study with two arms: a monocentric prospective study of patients evaluated for suspected endogenous glucocorticoid excess (cohort 1), and a multicenter cross-sectional study of patients in remission from CS, comparing those with recovered versus persistent adrenal insufficiency (cohort 2). All participants underwent standardised assessments and completed validated questionnaires on socioeconomic status, work ability, and fatigue.

**Results:**

In cohort 1, individuals with active CS (*n* = 28) and excluded CS (*n* = 56) had comparable comorbidities, education, and employment. However, work ability scores were lower in active CS (median 22.5 vs. 28.5, *p* = 0.008) and correlated inversely with biochemical cortisol excess. In multivariable analyses, active CS and depression were independently associated with lower work ability, whereas older age and depression were independently associated with fatigue severity. In cohort 2 (*n* = 89, 39 with recovered, 50 with persistent adrenal insufficiency), overall employment was 69%. Poor work ability was common (42% with recovered vs. 58% with persistent adrenal insufficiency) and weekly working hours were lower in those with persistent insufficiency (33 h vs. 39 h, *p* = 0.051). Overall, illness-related absences occurred in 76% during the preceding year, disability was recognised in 47%, and 15% received reduced earning capacity pensions. Fatigue correlated negatively with work ability (*r* = − 0.73, *p* < 0.0001).

**Conclusion:**

There is an unmet need for structured rehabilitation and reintegration in CS.

**Supplementary Information:**

The online version contains supplementary material available at https://doi.org/10.1007/s40618-026-02966-5.

## Introduction

Endogenous Cushing’s syndrome (CS) is a severe endocrine disorder due to prolonged exposure to excessive cortisol, leading to high morbidity and mortality [1–4]. Approximately 70% of cases result from ACTH-secreting pituitary adenomas, 20% from ACTH-independent adrenal disease, and 10% from ectopic ACTH production [5]. Diagnosing CS is challenging as symptoms are diverse and often overlooked or mistaken for lifestyle-related conditions [5–8]. Even after successful treatment, many patients experience persistent comorbidities and impaired quality of life, reflecting a sustained cardiometabolic and psychological burden [2, 9–12]. Socioeconomic consequences of CS are far less studied, with evidence mainly delivered from small monocentric cohorts. In Finland, less than half of 74 treated patients with CS reported full recovery, while 19% were permanently unable to return to work [13]. In the Netherlands, patients with pituitary and adrenal CS showed lower educational attainment, reduced employment, greater self-reported inability to work, and fewer relationships compared with matched controls despite being in long-term remission [14]. Moreover, in a cohort of 241 patients with different pituitary tumours, unemployment was highest among those with Cushing’s disease (CD, 47%) [15]. The European Registry of Cushing’s Syndrome (ERCUSYN) reported an unemployment rate of 14% and a sick leave rate of 9% in 2011 [16]. However, risk factors or underlying causes have not been investigated and more recent ERCUSYN data are not available. Nationwide register-based studies from Denmark and Sweden have further confirmed the long-term socioeconomic burden of CS. A Danish study of 424 patients with adrenal or pituitary CS showed persistently reduced employment from six years before to ten years after diagnosis, suggesting a diagnostic delay of 6 years [17], which is more than twice as long as previously observed by us [18]. Likewise, sick leave and disability pension were still elevated a decade after diagnosis, and even patients with favorable prognostic factors had reduced chances of full-time employment two years after surgery. Similarly, a Swedish study including 371 patients with Cushing’s disease reported declining employment and income and rising disability pensions, affecting 20–25% of patients during long-term follow-up. Disability pension rates were already increased six years before diagnosis and continued to rise thereafter, highlighting the substantial diagnostic delay and persistent socioeconomic burden despite remission [19]. However, both studies were register-based and the authors emphasised the lack of clinical data as the most important limitation of their studies.

To date, no socioeconomic or occupational data on patients with CS have been reported from Germany. Beyond addressing this geographic gap, studying these outcomes within the German healthcare and social security system is particularly relevant because disability assessment guidelines classify surgically treated patients with CS as “cured” [20] (see Supplement) potentially overlooking persistent functional limitations. We therefore aimed to evaluate objective parameters like educational level, employment status and disability pension, and work ability as a subjective measure in patients with newly diagnosed CS and those in remission, with a specific focus on the impact of persistent adrenal insufficiency. To complement the findings from aforementioned registry-based studies and to enhance interpretability, we incorporated detailed clinical and biochemical phenotyping.

## Methods

### Study setting and ethical approval

This was a national multicenter observational study in Germany with one prospective and one cross-sectional study arm. The lead study center, LMU Hospital Munich, is a reference center of the European Reference Network on Rare Endocrine Conditions (Endo-ERN). The study was conducted within the NeoExNet (Exzellenz-Netzwerk für Neuroendokrine Tumoren) registry and approved by the LMU Hospital Munich (study number 152-10) and by the ethic committee of the Berliner Ärztekammer (Eth-S-Q/14). It complies with the Declaration of Helsinki and all participating patients provided written informed consent. This study was reported in accordance with the STROBE (Strengthening the Reporting of Observational Studies in Epidemiology) guidelines (https://www.strobe-statement.org/).

### Patient recruitment and selection

Two distinct cohorts were included in this study: cohort 1 consisted of individuals prospectively evaluated for suspected endogenous glucocorticoid excess, and cohort 2 consisted of cross-sectionally enrolled patients with CS in remission. Cohort 1 was recruited in a single-center setting at the outpatient department of the LMU Hospital Munich. Based on standardised, guideline-recommended diagnostic procedures [21–23], patients were classified as having overt CS or no evidence of endogenous cortisol excess (“non-CS”). Importantly, the “non-CS” control group did not represent a healthy reference population but rather individuals presenting with similar clinical complaints suggestive of hypercortisolism. In these, CS was ruled out based on comprehensive clinical assessment by experienced endocrinologists and study nurses in our specialised Cushing’s outpatient department, combined with repeated biochemical testing including urinary free cortisol, late-night salivary cortisol, and the 1 mg dexamethasone suppression test. To minimise diagnostic uncertainty, individuals with an inconclusive diagnosis (e.g., discordant biochemical findings or incomplete diagnostic workup), patients with mild autonomous cortisol secretion (MACS), and patients receiving systemic glucocorticoid therapy were excluded. Cohort 2 was recruited in a multicenter setting from the outpatient department of the LMU Hospital Munich, an endocrinology practice in Berlin-Charlottenburg, and through public announcements on the German patient support platform *Glandula* (https://www.glandula-online.de/). At LMU Hospital Munich, approximately 25 patients are newly diagnosed with overt endogenous CS annually and around 200 patients with CS in remission are followed longitudinally. At the endocrinology practice in Berlin-Charlottenburg, approximately three new CS cases are diagnosed annually and around 40 patients in remission are followed regularly.

Remission was defined as either prolonged adrenal insufficiency or adequate suppression of morning serum cortisol (< 1.8 µg/dL) following a 1 mg dexamethasone suppression test, after tumour resection and/or bilateral adrenalectomy. Patients with CS in remission were divided into two groups for further analyses: those with recovery of the corticotroph axis (“CS-RAI” – Cushing’s syndrome with recovered adrenal insufficiency) and those with persistent adrenal insufficiency (“CS-PAI” – Cushing’s syndrome with persistent adrenal insufficiency). Recruitment occurred between October 2023 and February 2025, with most participants enrolled during the main recruitment phase from October 2023 to September 2024 (12 months).

### Data collection and questionnaires

Clinical and biochemical data were extracted from patient records by means of a pre-defined standardised data spreadsheet and collected locally at the LMU hospital Munich and the endocrinology practice Berlin-Charlottenburg. Patients recruited via the patient support platform *Glandula* were asked to share their medical records with the lead study team of the LMU Hospital Munich, where their data were medically reviewed and extracted. The following validated questionnaires were used: The KORA (“Cooperative Health Research in the Region of Augsburg” study) questionnaire for standardised assessment of education, family and employment status, and income [24, 25]; the Work Ability Index (WAI) for assessment of subjective work ability within the past four weeks [26, 27]; as well as the Fatigue Severity Scale to assess symptoms related to fatigue [28–30]. Patients could choose between answering questionnaires via the online web platform REDCap or paper based.

### Statistical analysis

Statistical analyses were performed using GraphPad Prism, version 10.1.1 and R version 4.4.2. Continuous variables were reported as medians with interquartile ranges (IQR) and categorical variables as percentages alongside the number of available data. The Mann-Whitney *U* test was used for comparisons between two unpaired continuous variables. Binary outcomes were analyzed using Fisher’s exact test. Associations between variables were examined using Spearman correlation and simple linear regression. Exploratory complete-case multiple linear regression analyses adjusted for diagnostic group, age, sex, BMI, diabetes mellitus type 2, and depression were performed to assess the effect of comorbidities on WAI and fatigue severity. A two-sided p-value of < 0.05 was considered statistically significant. As this was an exploratory study, no adjustments were made for multiple comparisons.

## Results

### Overview of recruited patients per center (cohorts 1 and 2)

**Fig. 1:**
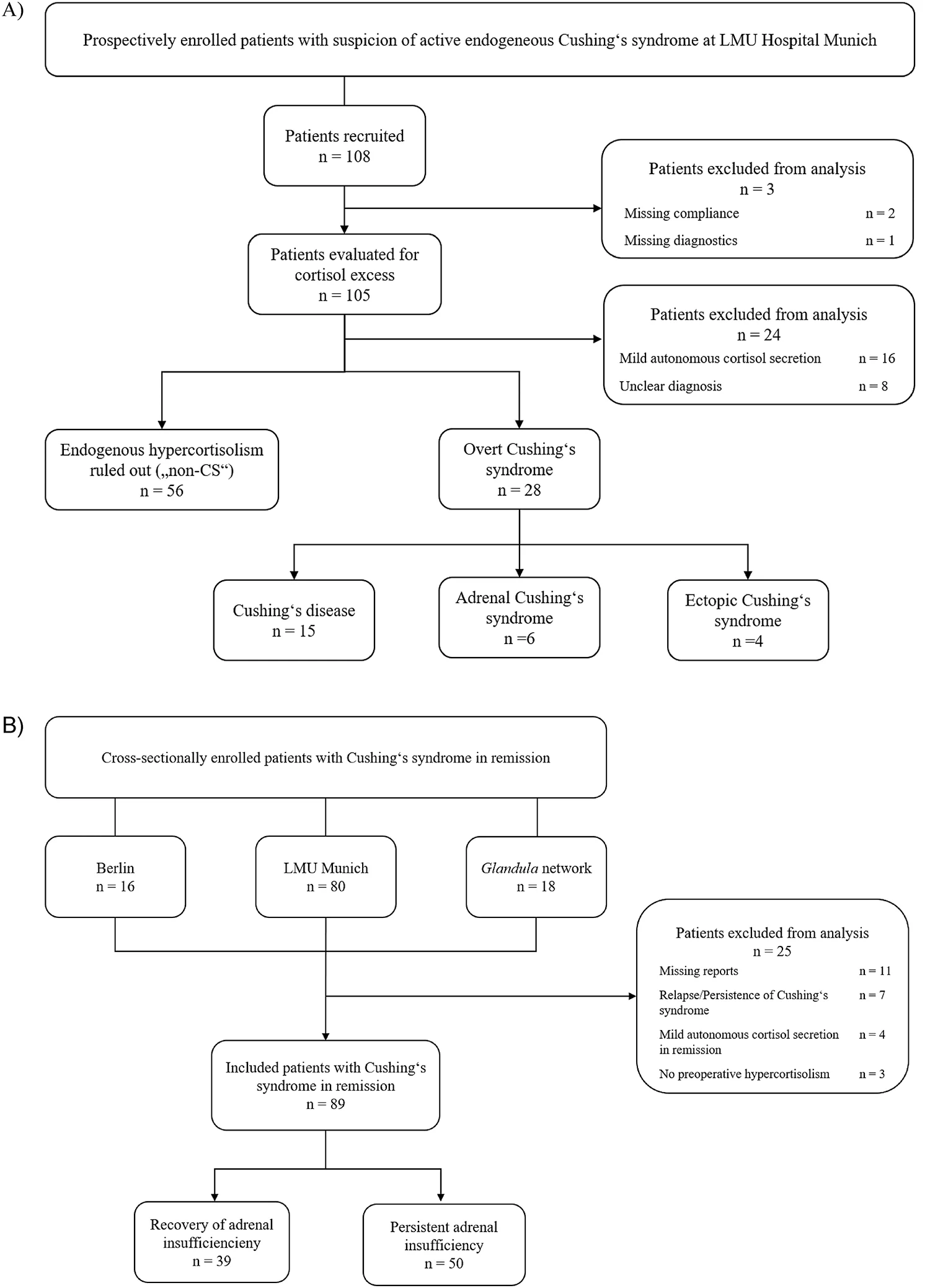
Patient enrolment and subtyping of cohort 1 (**A**) and cohort 2 (**B**)

Across all centers, a total of 225 participants completed the questionnaires, of whom 197 met the inclusion criteria (Fig. 1 provides the number of participants contributed by each center and details of exclusions). In cohort 1, 108 individuals were prospectively screened for endogenous glucocorticoid excess at the LMU Hospital (Fig. 1A). Among these, overt CS was confirmed in 28 patients, while 56 individuals had exclusion of CS and served as reference group, and the remaining did not meet the inclusion criteria. In cohort 2, after applying exclusion criteria, 66 patients from the LMU hospital, 16 from the endocrinology outpatient clinic Berlin-Charlottenburg, and 7 recruited via the *Glandula* network were included, resulting in a total of 89 patients with CS in remission (Fig. 1B).

### Occupational health, work ability, and fatigue in prospectively evaluated individuals (cohort 1)

**Fig. 2:**
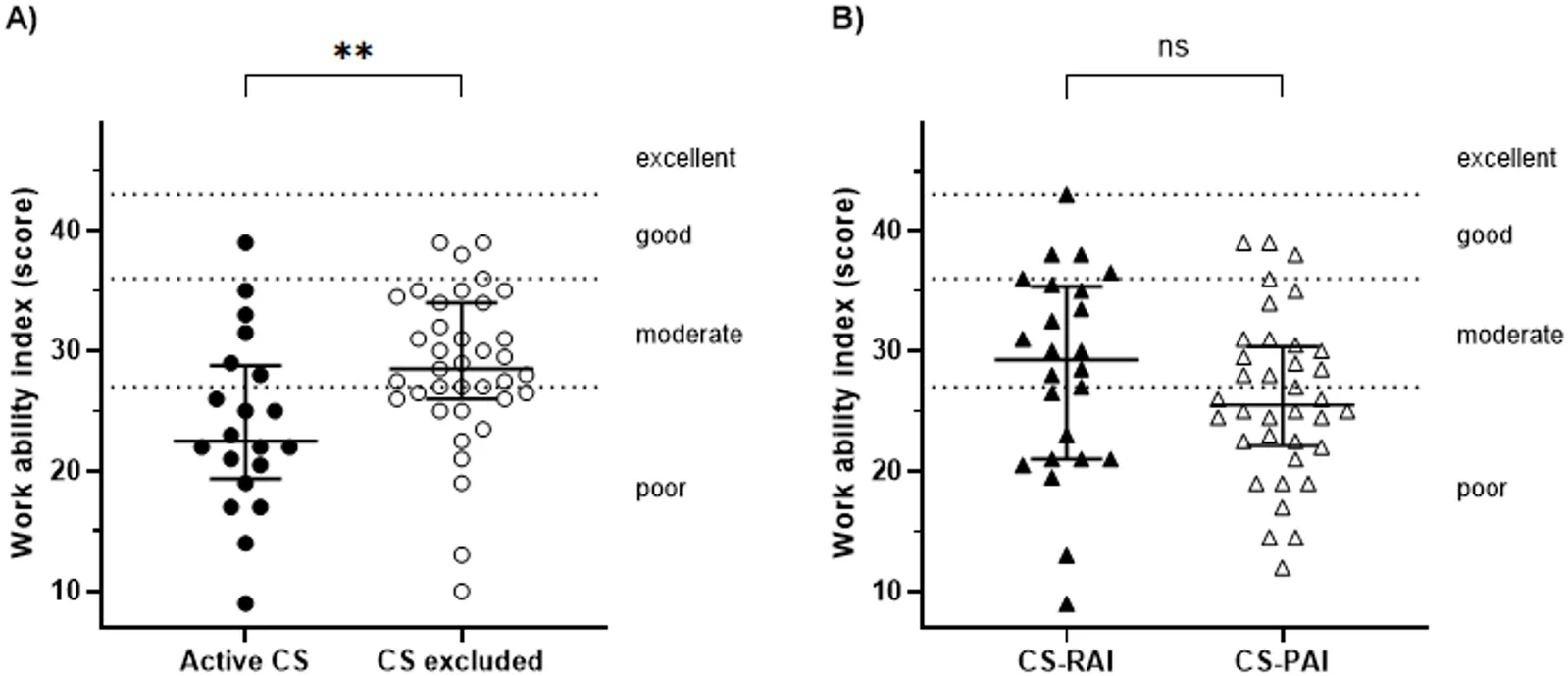
Work ability index in employed patients in cohort 1 (**A**) and 2 (**B**). Higher scores indicate better subjective work ability. Work ability was analyzed only in patients who were employed at questionnaire completion and provided complete data (active CS, *n* = 20; non-CS, *n* = 37; CS-RAI, *n* = 24; CS-PAI, *n* = 36). The reference cohort of individuals with excluded Cushing’s syndrome (**A**) does not represent a healthy control cohort but predominantly of overweight individuals with metabolic disease. Mann-Whitney-U test for pairwise comparisons (active CS vs. non-CS: *p* = 0.008; CS-RAI vs. CS-PAI: *p* = 0.25; active CS vs. CS-RAI: *p* = 0.074; active CS vs. CS-PAI: *p* = 0.235; non-CS vs. CS-RAI: *p* = 1.00; non-CS vs. CS-PAI: *p* = 0.057). For clarity, only key comparisons are shown in the figures. CS, Cushing’s syndrome; CS-RAI, Cushing’s syndrome with recovered adrenal insufficiency; CS-PAI, Cushing’s syndrome with persistent adrenal insufficiency; non-CS, Cushing’s syndrome excluded; ns, not statistically significant; **p < 0.01

**Fig. 3:**
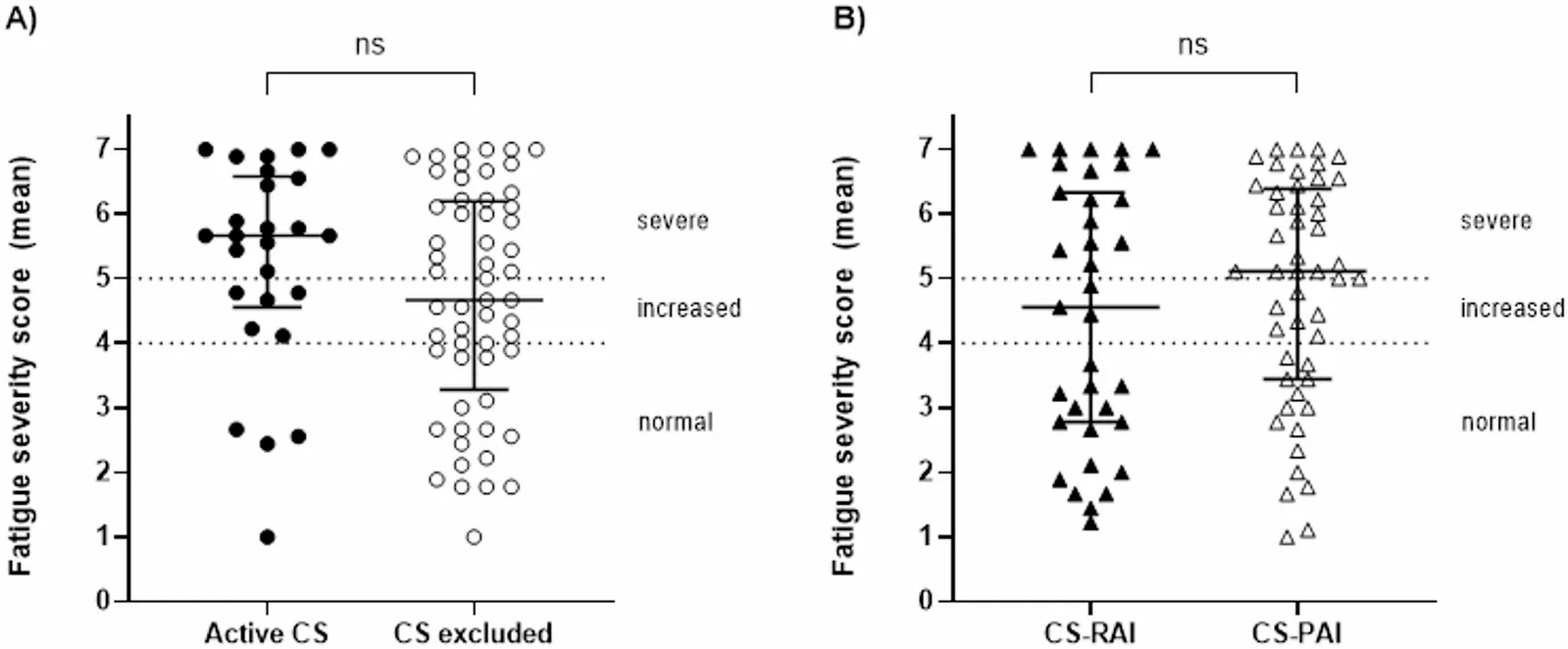
Fatigue severity score in cohort 1 (**A**) and 2 (**B**). Higher scores indicate more severe fatigue. The reference cohort of individuals with excluded Cushing’s syndrome (**A**) does not represent a healthy control cohort but predominantly of overweight individuals with metabolic disease. Mann-Whitney-U test for pairwise comparisons (not significant (ns) for all two-group comparisons across the entire study cohort). CS, Cushing’s syndrome; CS-RAI, Cushing’s syndrome with recovered adrenal insufficiency; CS-PAI, Cushing’s syndrome with persistent adrenal insufficiency

Baseline characteristics of individuals prospectively screened for CS are shown in Table 1. Of those diagnosed with overt CS (*n* = 28), 15 had CD (54%), 9 adrenal CS (32%), and 4 ectopic CS (14%). Patients with confirmed and ruled-out CS were similar in age (median age in CS: 42.0 years [32.3–59.0] vs. non-CS 40.0 [31.0–55.8]; *p* = 0.53) and sex distribution (females among CS: 89% vs. non-CS 79%; *p* = 0.37). Typical CS-associated comorbidities were present in both groups, with higher rates of type 2 diabetes in confirmed CS (29% vs. 5%, *p* = 0.005). Notably, the non-CS group did not represent healthy controls but predominantly consisted of overweight individuals with metabolic disease. Sociodemographic and occupational characteristics are summarised in Table 2. Patients with newly diagnosed CS more often lived with a partner (78% vs. 48%, *p* = 0.011) but had similar educational attainment and employment rates as non-CS. Weekly working hours tended to be lower in CS (30 vs. 39 h, *p* = 0.071). Work ability was markedly reduced (median WAI score 22.5 vs. 28.5, *p* = 0.008, Fig. 2A), with 70% of working patients with CS rating their work ability as “poor” compared with 41% of non-CS (*p* = 0.034). Illness-related absence from work within the past year was common in both groups (69% vs. 79%, *p* = 0.41), with similar durations of sick leave. A recognised disability was reported in 26% of patients with CS and 23% of non-CS; severe disability (≥ 50%) was more frequent in CS (71% vs. 54%, *p* = 0.64). Fatigue scores were slightly higher in CS but not statistically different (*p* = 0.13; Fig. 3A).

**Table 1:** Clinical characteristics in prospectively enrolled patients with confirmed or ruled-out Cushing’s syndrome (cohort 1)

	Active Cushing’s syndrome	Cushing’s syndrome excluded	*p*-value
*n*	28	56	-
**Demographic characteristics**			-
Age in years, median (IQR)	42.0 (32.3–59.0)	40.0 (31.0–55.8)	0.53
Female sex, n/n (%)	25/28 (89)	44/56 (79)	0.37
BMI in kg/m^2^, median (IQR)	29.0 (25.0–35.3)	29.3 (24.6–33.4)	0.89
**Relationship status**			-
Married/ long term partnership, n/n (%)	18/27 (67)	25/56 (45)	0.060
Shared household with partner, n/n (%)	21/27 (78)	27/56 (48)	**0.011**
**Tumour localisation**			-
Pituitary, n/n (%)	15/28 (54)	-	-
Adrenal, n/n (%)	9/28 (32)	-	-
Ectopic, n/n (%)	4/28 (14)	-	-
**Biochemical evaluation**			-
Serum cortisol in µg/dL, median (IQR)	21.3 (14.1–29.7)	7.6 (4.8–10.8)	**< 0.001**
Serum ACTH in pg/mL, median (IQR)	53.4 (9.0–72.5)	11.0 (8.0–15.0)	**< 0.001**
UFC in µg/24 h, median (IQR)	416 (175–681)	80.3 (61.6–119)	**< 0.001**
LNSC in ng/mL, median (IQR)	11.4 (6.3–16.5)	1.9 (1.2–2.8)	**< 0.001**
1-mg DST in µg/dL, median (IQR)	13.0 (8.8–20.0)	1.0 (0.7–1.4)	**< 0.001**
**Comorbidities at current visit**			-
Diabetes type 2, n/n (%)	8/28 (29)	3/56 (5)	**0.005**
Pathological glucose tolerance, n/n (%)	8/28 (29)	8/56 (14)	0.12
Arterial hypertension, n/n (%)	16/28 (57)	31/56 (55)	0.88
Osteoporosis, n/n (%)	1/28 (4)	3/56 (5)	1.0
Pathological fractures, n/n (%)	0/28 (0)	1/56 (2)	1.0
Severe or opportunistic infections requiring hospitalization, n/n (%)	1/28 (4)	1/56 (2)	1.0
Thromboembolic events, n/n (%)	2/28 (7)	1/56 (2)	0.26
Obstructive sleep apnea, n/n (%)	4/28 (14)	3/56 (5)	0.22
Depression, n/n (%)	3/28 (11)	7/56 (13)	1.0
Active or former smoker, n/n (%)	12/28 (43)	24/66 (36)	0.64

The reference cohort of individuals with excluded Cushing’s syndrome does not represent a healthy control cohort but predominantly of overweight individuals with metabolic disease. Mann-Whitney U for comparisons between two unpaired continuous variables, Fisher’s exact test for binary outcomes

ACTH, Adrenocorticotropic Hormone; BMI, Body Mass Index; CS, Cushing’s syndrome; DST, Dexamethasone Suppression Test IQR, Interquartile Range; LNSC, Late Night Salivary Cortisol; UFC, Urinary Free Cortisol

**Table 2:** Social and occupational aspects in pension in prospectively enrolled patients with confirmed or ruled-out Cushing’s syndrome (cohort 1)

	Active Cushing’s syndrome	Cushing’s syndrome excluded	*p*-value
*n*	28	56	-
**Education**			**-**
University entrance certificate or higher level of education, n/n (%)	13/27 (48)	25/56 (45)	0.76
ISCED low (0–2)	4/27 (15)	7/56 (13)	-
ISCED medium (3–4)	13/27 (48)	31/56 (55)	-
ISCED high (5–8)	10/27 (37)	18/56 (32)	-
**Current employment status**			0.62*
Retirement, pension, n/n (%)	4/27 (15)	8/55 (15)	-
Passive phase of partial retirement, n/n (%)	0/27 (0)	0/55 (0)	-
Active phase of partial retirement, n/n (%)	0/27 (0)	0/55 (0)	-
Employed, n/n (%)	21/27 (78)	40/55 (73)	-
Registered as unemployed, n/n (%)	0/27 (0)	3/55 (5)	-
Other, n/n (%)	2/27 (7)	4/55 (7)	-
**Current or previous main employment type**			0.049**
Worker, n/n (%)	2/27 (7)	3/55 (5)	-
Employee, n/n (%)	18/27 (67)	47/55 (85)	-
Civil servant, n/n (%)	5/27 (19)	0/55 (0)	-
Self-employed, n/n (%)	2/27 (7)	3/55 (5)	-
Helping Family member, n/n (%)	0/27 (0)	0/55 (0)	-
Other, n/n (%)	0/27 (0)	2/55 (4)	-
**Working hours**			
Contractual weekly working hours of employed individuals, median (IQR)	30.0 (24.8–40.0)	39.0 (30.0–40.0)	0.07
Working days per week of employed individuals, median (IQR)	5 (4–5)	5 (5–5)	0.084
**Sick-leave**			
Illness-related absence of work during the past 12 months, yes, n/n (%)	18/26 (69)	41/52 (79)	0.41
Estimated number of days on sick leave during the past 12 months, median (IQR)	23.0 (9.5–118)	24.5 (7.8–72.5)	0.90
**Reduced earning capacity pension**			
Reception of a reduced earning capacity pension, n/n (%)	2/27 (7)	4/56 (7)	1.0
**Disability status**			
Presence of a recognised disability, n/n (%)	7/27 (26)	13/56 (23)	0.79
Degree of disability < 50, n/n (%)	2/7 (29)	6/13 (46)	0.64
Degree of disability ≥ 50, n/n (%)	5/7 (71)	7/13 (54)	0.64

The reference cohort of individuals with excluded Cushing’s syndrome does not represent a healthy control cohort but predominantly of overweight individuals with metabolic disease. Mann-Whitney U for comparisons between two unpaired continuous variables, Fisher’s exact test for binary outcomes

CS, Cushing’s syndrome; ISCED, International Standard Classification of Education; IQR, interquartile range

*Employed versus all other categories

**Employee versus all other categories

### Occupational health, work ability, and fatigue in patients with CS in remission (cohort 2)

Baseline characteristics of patients with CS in remission are summarised in Table 3. Thirty-nine patients were classified as CS-RAI and 50 as CS-PAI. Groups were comparable in age, sex, comorbidities, and CS etiology (overall: 70% CD, 26% adrenal CS, 5% ectopic CS). Permanent adrenal insufficiency following bilateral adrenalectomy accounted for 36% of CS-PAI. Median remission duration was 7.2 years and similar between groups. Sociodemographic characteristics were also comparable (Table 4): 62% lived with a partner, and ~ 50% in each group had a university entrance qualification or higher. Despite being of working age, employment rates were modest (69%), with a trend toward more premature retirement in CS-RAI (26% vs. 10%, *p* = 0.085). Among employed patients, median weekly working hours were lower in CS-PAI vs. CS-RAI (33 vs. 39 h, *p* = 0.051). Overall, work ability was mostly rated as poor or moderate (52% and 37%, respectively), with slightly though not statistically significantly lower scores in CS-PAI (median WAI score 25.5 vs. 29.3, *p* = 0.25, Fig. 2B). Illness-related work absence during the past year was common in both groups (CS-RAI: 69% vs. CS-PAI: 81%; *p* = 0.30), with comparable durations of sick leave. Overall, 15% of patients received a reduced earning-capacity pension. A recognised disability was reported in 47% of the cohort, and 51% of these were classified as severely disabled (degree of disability ≥ 50), with slightly higher frequencies in CS-PAI. Increased or severe fatigue was reported by 63% of patients, without relevant differences across groups (Fig. 3B). Among patients with CS-PAI, no significant differences in work ability or fatigue severity were observed between those who had undergone bilateral adrenalectomy (*n* = 18) and those who had not (*n* = 32). Median WAI scores were 25.5 (IQR 20.5–35.3) in patients after bilateral adrenalectomy versus 25.5 (IQR 21.3–30.1) in those without bilateral adrenalectomy (*p* = 0.76). Similarly, fatigue severity scores did not differ significantly between groups (4.6 [IQR 2.9–6.5] vs. 5.2 [IQR 3.7–6.3], *p* = 0.45).

**Table 3:** Clinical characteristics in cross-sectionally enrolled patients with Cushing’s syndrome in remission (cohort 2)

	CS in remission, total	CS-RAI	CS-PAI	*p*-value
*n*	89	39	50	-
**Demographic characteristics**				-
Age in years, median (IQR)	52.0 (42.0–61.5)	56.0 (39.0–64.0)	51.0 (42.0–58.3)	0.34
Female sex, n/n (%)	73/89 (82)	32/39 (82)	41/50 (82)	1.0
BMI in kg/m^2^, median (IQR)	26.5 (23.5–30.9)^a^	27.6 (23.5–30.2)	25.8 (23.4–31.1)	0.55
**Relationship status**				-
Married/ long term partnership, n/n (%)	50/88 (57)	19/38 (50)	31/50 (62)	0.29
Shared household with partner, n/n (%)	55/89 (62)	23/39 (59)	32/50 (64)	0.67
**Tumour localisation**				0.59
Pituitary, n/n (%)	62/89 (70)	25/39 (64)	37/50 (74)	-
Adrenal, n/n (%)	23/89 (26)	12/39 (31)	11/50 (22)	-
Ectopic, n/n (%)	4/89 (5)	2/39 (5)	2/50 (4)	-
**Treatment modality**				-
Pituitary surgery, n/n (%)	63/89 (71)	26/39 (67)	37/50 (74)	0.35
Successful, n/n (%)	47/63 (75)	22/26 (85)	25/37 (68)	0.15
Unilateral Adrenalectomy, n/n (%)	20/89 (22)	11/39 (28)	9/50 (18)	0.31
Bilateral Adrenalectomy, n/n (%)	19/89 (21)	1/39 (3)^b^	18/50 (36)	**< 0.001**
Ectopic tumour surgery, n/n (%)	2/89 (2)	2/39 (5)	0/50 (0)	0.19
Successful, n/n (%)	2/2 (100)	2/2 (100)	0/0 (0)	1.0
Tumour radiation, n/n (%)	11/89 (12)	3/39 (8)	8/50 (16)	0.34
Time since remission in years, median (IQR)	7.2 (2.7–16.9)	7.2 (3.5–17.2)	6.8 (1.9–15.9)	0.43
**Pituitary insufficiency**				-
Corticotropic axis insufficiency, n/n (%)	52/89 (58)	0/39 (0)	50/50 (100)	**0.001**
Substituted, n/n (%)	50/89 (56)	0/39 (0)	50/50 (100)	-
Somatotropic axis insufficiency, n/n (%)	9/89 (10)	3/39 (8)	6/50 (12)	0.73
Substituted, n/n (%)	4/89 (4)	1/39 (3)	3/50 (6)	-
Gonadotropic axis insufficiency, n/n (%)	9/89 (10)	4/39 (10)	5/50 (10)	1.0
Substituted, n/n (%)	3/89 (3)	1/39 (3)	2/50 (4)	-
Thyrotropic axis insufficiency, n/n (%)	7/89 (8)	3/39 (8)	4/50 (8)	1.0
Substituted, n/n (%)	6/89 (7)	2/39 (5)	4/50 (8)	-
Posterior pituitary gland dysfunction/ ADH deficiency, n/n (%)	1/89 (1)	0/39 (0)	1/50 (2)	1.0
Substituted, n/n (%)	1/89 (1)	0/39 (0)	1/50 (2)	-
**Comorbidities at current visit**				-
Diabetes, n/n (%)	14/89 (16)	7/39 (18)	7/50 (14)	0.77
Pathological glucose tolerance, n/n (%)	8/73 (11)	6/33 (18)	2/40 (5)	0.13
Arterial hypertension, n/n (%)	34/89 (38)	19/39 (49)	15/50 (30)	0.083
Osteoporosis, n/n (%)	22/88 (25)	6/39 (15)	16/49 (33)	0.084
Pathological fractures, n/n (%)	9/88 (10)	4/39 (10)	5/49 (10)	1.0
Severe or opportunistic infections requiring hospitalization, n/n (%)	6/89 (7)	1/39 (3)	6/50 (12)	0.23
Thromboembolic events, n/n (%)	7/88 (8)	2/39 (5)	5/49 (10)	0.46
Obstructive sleep apnea, n/n (%)	4/88 (5)	2/39 (5)	2/49 (4)	1.0
Depression, n/n (%)	14/88 (16)	6/39 (15)	8/49 (16)	1.0
Active or former smoker, n/n (%)	24/68 (35)	12/31 (39)	12/37 (32)	0.62

Mann-Whitney U for comparisons between two unpaired continuous variables, Fisher’s exact test for binary outcomes

ADH, Antidiuretic Hormone; BMI, Body Mass Index; CS, Cushing’s syndrome; CS-PAI, Cushing’s syndrome with persistent adrenal insufficiency; CS-RAI, Cushing’s syndrome with recovered adrenal insufficiency; HC, Hydrocortisone; IQR, Interquartile Range

^a^Values available for 38 patients with CS in remission without HC replacement and 48 patients with CS in remission with HC replacement

**Table 4:** Social and occupational aspects in cross-sectionally enrolled patients with Cushing’s syndrome in remission (cohort 2)

	CS in remission, total	CS-RAI	CS-PAI	*p*-value
*n*	89	39	50	-
**Education**				**-**
University entrance certificate or higher level of education, n/n (%)	45/86 (52)	19/37 (51)	26/49 (53)	0.88
ISCED low (0–2)	6/86 (7)	1/37 (4)	5/49 (10)	-
ISCED medium (3–4)	50/86 (58)	21/37 (57)	29/49 (59)	-
ISCED high (5–8)	30/86 (35)	15/37 (41)	15/49 (31)	-
**Current employment status**				0.061*
Retirement, pension, n/n (%)	15/88 (17)	10/39 (26)	5/49 (10)	-
Passive phase of partial retirement, n/n (%)	3/88 (3)	2/39 (5)	1/49 (2)	-
Active phase of partial retirement, n/n (%)	1/88 (1)	0/39 (0)	1/49 (2)	-
Employed, n/n (%)	61/88 (69)	23/39 (59)	38/49 (78)	-
Registered as unemployed, n/n (%)	2/88 (2)	1/39 (3)	1/49 (2)	-
Other, n/n (%)	6/88 (7)	3/39 (8)	3/49 (6)	-
**Current or previous main employment type**				0.33**
Worker, n/n (%)	3/87 (3)	1/37 (3)	2/50 (4)	-
Employee, n/n (%)	70/87 (80)	28/37 (76)	42/50 (84)	-
Civil servant, n/n (%)	5/87 (6)	3/37 (8)	2/50 (4)	-
Self-employed, n/n (%)	6/87 (7)	4/37 (11)	2/50 (4)	-
Helping Family member, n/n (%)	1/87 (1)	1/37 (3)	0/50 (0)	-
Other, n/n (%)	2/87 (2)	0/37 (0)	2/50 (4)	-
**Working hours**				
Contractual weekly working hours of employed individuals, median (IQR)	34.7 (25.0–39.0)	38.5 (30.0–40.0)	32.5 (25.0–38.5)	0.089
Working days per week of employed individuals, median (IQR)	5 (5–5)	5 (5–5)	5 (5–5)	0.70
**Sick-leave**				
Illness-related absence of work during the past 12 months, yes, n/n (%)	63/83 (76)	25/36 (69)	38/47 (81)	0.30
Estimated number of days on sick leave during the past 12 months, median (IQR)	18.5 (9.3–49.8)	14.5 (9.0–45.0)	19.5 (10.0–52.8)	0.68
**Reduced earning capacity**				
Reception of a reduced earning capacity pension, n/n (%)	13/87 (15)	7/37 (19)	6/50 (12)	0.38
**Disability**				
Presence of a recognised disability, n/n (%)	41/87 (47)	14/37 (38)	27/50 (54)	0.19
Degree of disability < 50, n/n (%)	20/41 (49)	8/14 (57)	12/27 (44)	0.52
Degree of disability ≥ 50, n/n (%)	21/41 (51)	6/14 (43)	15/27 (56)	0.52

Mann-Whitney U for comparisons between two unpaired continuous variables, Fisher’s exact test for binary outcomes

CS, Cushing’s syndrome; CS-PAI, Cushing’s syndrome with persistent adrenal insufficiency; CS-RAI, Cushing’s syndrome with recovered adrenal insufficiency; ISCED, International Standard Classification of Education; IQR, interquartile range; HC, hydrocortisone; CS-PAI, Cushing’s syndrome with Persistent Adrenal Insufficiency; CS-RAI, Cushing’s syndrome with Recovery of Adrenal Insufficiency

*Employed versus all other categories

**Employee versus all other categories

### Factors associated with worse work ability

**Fig. 4:**
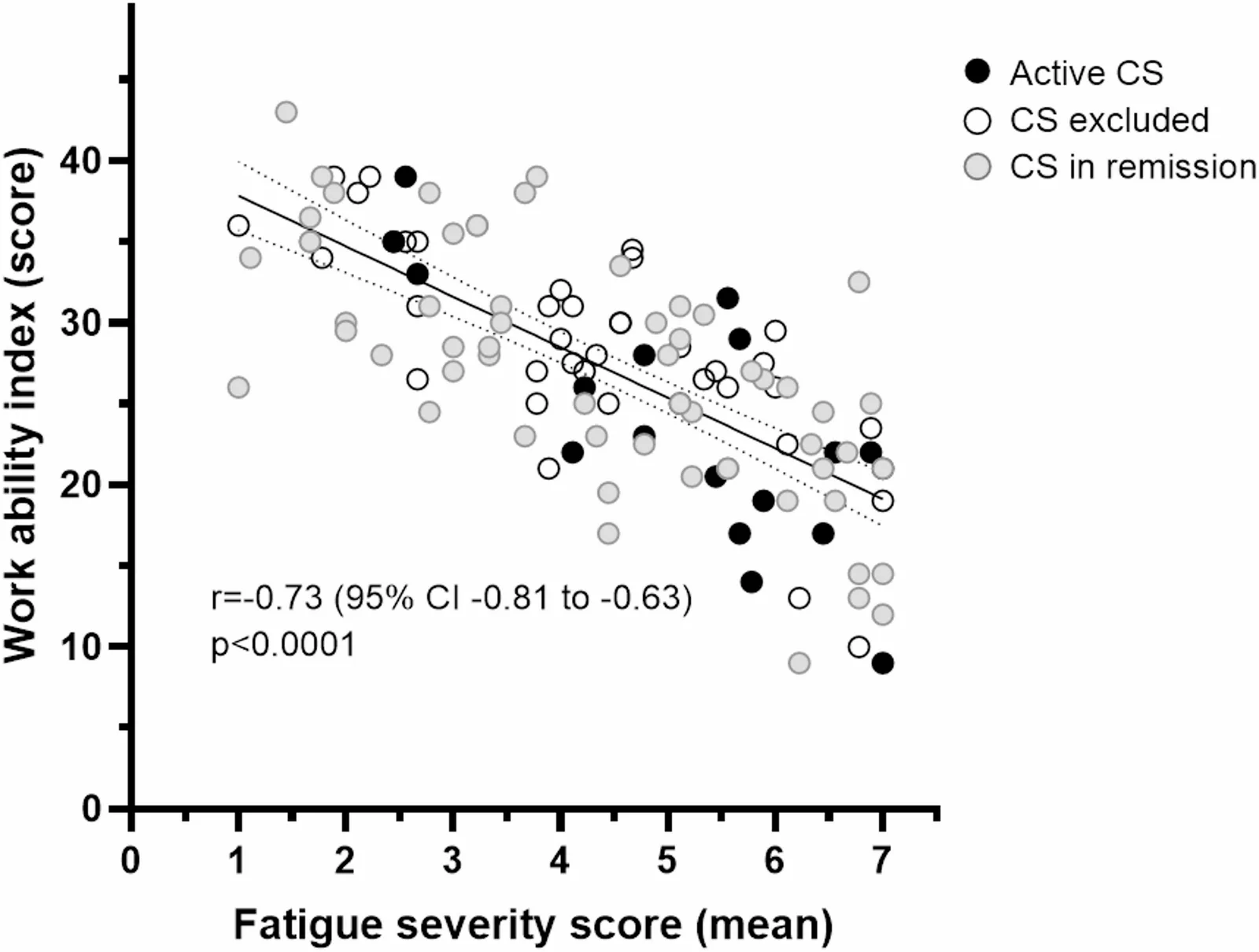
Correlation of work ability and fatigue severity in the entire study cohort. Graph depicts Spearman correlation with linear regression curve and 95% CI-intervals of the entire study cohort (i.e., patients during active disease, during remission and individuals with excluded CS). Again, work ability was analyzed only in patients who were employed at questionnaire completion and provided complete data. Patients with active CS are highlighted in black. CS, Cushing’s syndrome

Due to the study design, individual prognostic factors for long-term occupational-health related outcomes could not be investigated. However, we were able to assess associations between biochemical and clinical parameters and subjectively rated work ability. Indeed, in the prospective study arm (i.e., active CS and non-CS), we observed a weak to moderate inverse correlation between WAI and the degree of cortisol excess measured by UFC (*r* = − 0.39 [95% CI − 0.61 to − 0.13], *p* = 0.004), LNSC (*r* = − 0.38 [95% CI − 0.60 to − 0.11], *p* = 0.006) and morning serum cortisol following 1 mg DST (*r* = − 0.29 [95% CI − 0.52 to − 0.02], *p* = 0.029). Likewise, fatigue severity also showed a weak positive correlation with morning serum cortisol following the baseline 1 mg DST (*r* = 0.27 [95% CI 0.05 to 0.46], *p* = 0.016), but not with other parameters of cortisol excess (data not shown). Moreover, among the entire study population, as well as among all individual subgroups there was a strong inverse correlation between fatigue severity and work ability (*r* = − 0.73 [95% CI − 0.81 to − 0.63, *p* < 0.0001; Fig. 4).

**Fig. 5:**
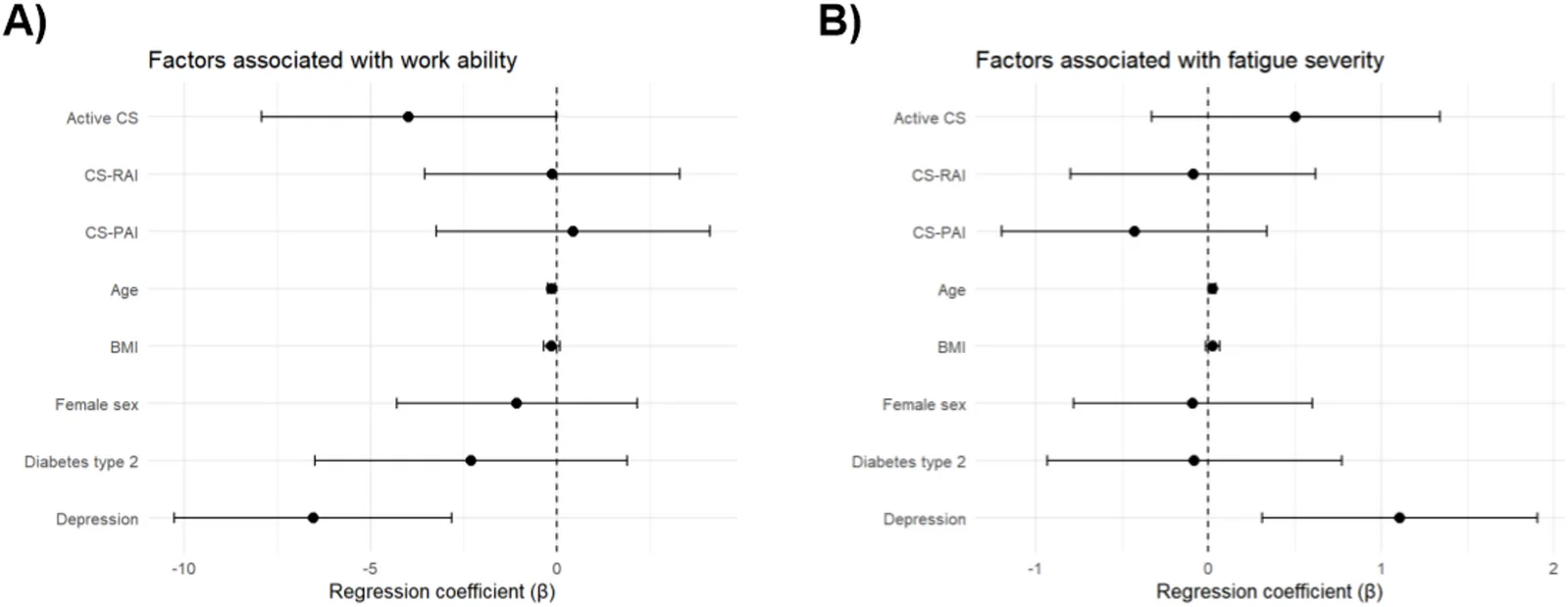
Factors associated with work ability and fatigue severity in patients with suspected or confirmed Cushing’s syndrome. Forest plots showing regression coefficients (β) and 95% confidence intervals from multivariable linear regression analyses assessing factors associated with (**A**) work ability and (**B**) fatigue severity. Models were adjusted for diagnostic group, age, sex, BMI, diabetes type 2, and depression. BMI, body mass index; CS, Cushing’s syndrome; CS-PAI, Cushing’s syndrome with persistent adrenal insufficiency; CS-RAI, Cushing’s syndrome with recovered adrenal insufficiency

To investigate the effect of comorbidities on WAI and fatigue severity, we performed an exploratory multiple linear regression analyses adjusted for diagnostic group, age, sex, BMI, diabetes type 2, and depression. Further pituitary deficiencies were not included as a covariate due to the low number of affected patients in the entire study cohort (*n* = 13). Active CS was independently associated with lower WAI compared with individuals in whom CS had been excluded (β = − 3.97, 95% CI − 7.93 to -0.01, *p* = 0.049). Older age (β = − 0.14, 95% CI − 0.24 to − 0.03, *p* = 0.011) and depression (β = − 6.54, 95% CI − 10.26 to − 2.81, *p* < 0.001) were also independently associated with lower WAI. BMI, sex, and diabetes type 2 were not significantly associated with either outcome (Fig. 5A). In the corresponding model for fatigue severity, diagnostic group did not show a significant association. However, older age (β = 0.024, 95% CI 0.004 to 0.04, *p* = 0.019) and depression (β = 1.11, 95% CI 0.31 to 1.90, *p* = 0.007) were independently associated with higher fatigue severity scores (Fig. 5B).

## Discussion

This multicenter study provides the first comprehensive evaluation of social and occupational health outcomes in CS in relation to detailed clinical and biochemical disease characterisation. We observed low subjective work ability, reduced weekly working hours, and high rates of disability in patients with both active and remitted disease.

Patients with newly diagnosed CS were generally well educated with 85% having a medium or high educational level. These rates are similar to reports on educational status from patients with CD in Sweden (81% ISCED medium or high) [19] and Denmark (66% ISCED medium or high) [17], and indicate that their “starting conditions” into working life were generally good. Despite higher employment rates at diagnosis (78%) compared to the Danish study (59%) [17], working hours in our cohort were significantly reduced and subjective work ability was rated poor. The WAI is a practical screening tool to evaluate how well an individual perceives their ability to meet the demands of their job in relation to their health and personal resources [27]. Consequently, there is no national comparative norm data available, and it is rather used to detect trends, monitor risk and help guide targeted interventions to recover work ability. It is important to highlight that the reference cohort of individuals with suspected but ruled-out CS also exhibited a substantial occupational health burden. This finding clearly suggests that adverse occupational and psychosocial outcomes are not solely attributable to cortisol excess (as also reflected by the overall weak-moderate correlation between cortisol excess and WAI) but are instead shaped by multifactorial determinants. Individuals referred for evaluation of endogenous CS at specialised endocrine and pituitary clinics often have a prolonged history of consulting multiple physicians. While these patients – many of whom present with non-neoplastic hypercortisolism – do not meet criteria for disease-specific interventions at such centers, it is crucial to raise awareness among clinicians about these frequently overlooked aspects. Identification of individuals with reduced work ability, or those at risk thereof (e.g., those with depression), should consistently prompt consideration of structured rehabilitation planning.

The observed impairment in work ability is likely related to the well-documented long-term effects of CS on patients’ health. For example, we reported that muscle function in patients with CS in remission remains impaired in the long term [31, 32]. Furthermore, treated patients with Cushing’s disease also show also increased hospitalization rates for psychiatric disorders and a trend toward higher rates of sepsis compared with patients with nonfunctioning pituitary adenomas after pituitary surgery [33]. In general, patients with Cushing’s disease who have been in remission for more than 10 years are still at increased risk of overall mortality compared with the general population, particularly due to circulatory diseases [4].

In line with previous studies [17, 19], we found lower employment rates among patients with CS in remission compared with patients during active disease, although the cross-sectional design in the present study impeded individual follow-up comparison. Notably, 47% of patients with CS in remission had a recognised disability, of which 51% were classified as being severe. These findings are particularly striking in the context of the current German disability assessment framework, which considers surgically treated patients with CS as “cured” and therefore typically not eligible for disability recognition [20] (see Supplement). Our data therefore reveals a clear mismatch between this formal classification and patients’ actual functional impairment, indicating a need to revise assessment criteria to better reflect the lasting occupational and health-related limitations in this population. Reduced earning capacity pension rates were slightly lower (15% at a median of 7.2 years since remission) compared to reported disability pension rates in patients with CD in remission from Sweden (25% at 9 years since remission) and Denmark (34% at 10 years since remission), although definitions and regulatory conditions across countries may vary. Working hours and subjective work ability were rated slightly, though not statistically significantly, worse amongst patients with persistent adrenal insufficiency compared with those in remission thereof. This trend may reflect the effect of unphysiological glucocorticoid replacement on energy or stress tolerance [34, 35], but larger studies are needed to confirm this. To our knowledge, no prior data on subjective work ability in patients with adrenal insufficiency exists. However, a recent study showed increased work loss in patients with Addison’s disease compared with matched controls [36], supporting a potential occupational impact of cortisol deficiency [37].

Fatigue is one of the most frequent as well as most bothersome symptoms reported by patients with CS [38]. Almost half of patients with treated CS still reported persistent sleep disruption with associations to ongoing mood disturbances, and one third required sleep medications [39]. In our study, we observed a high prevalence of fatigue across the entire cohort, with symptoms being most pronounced during the active disease phase and showing a strong association between fatigue severity and subjective work ability. Although these findings are not unexpected, they highlight the need for structured rehabilitation programs that specifically address fatigue and support reintegration into the workplace, considering individual psychological and physical resources as well as overall coping capacity. Fatigue is highly prevalent across a wide range of chronic diseases and is now widely acknowledged as a multidimensional phenomenon encompassing both psychological and physical aspects. While specific or causative treatment options remain limited, a recent systematic review and metanalysis in patients with long COVID demonstrated a substantial beneficial effect of transcranial direct current stimulation on fatigue [40]. Whether this intervention may also hold potential for patients with CS warrants further investigation.

Strengths of this study include its multicenter design including recruitment via a patient advocacy group and the combined prospective and cross-sectional arms, which will enable future longitudinal follow-up studies. Additional strengths are the use of validated questionnaires only, standardised guideline-based diagnostic procedures, and a nearly complete clinical and biochemical dataset, minimising the risk of diagnostic misclassification and overcoming limitations from register-based studies. Further strengths relate to the control cohort of prospectively enrolled patients with suspected, but biochemically ruled-out CS, in whom clinical appearance and comorbidities were similar. At the same time, this comparator group did not represent a healthy reference population. Consequently, the observed differences between groups were likely attenuated, and the true impact of active CS on occupational functioning may be substantially greater when compared with the general healthy population. Moreover, although individuals with inconclusive findings were excluded, mild or cyclic hypercortisolism cannot be fully excluded in all cases of those classified as “non-CS”. Further limitations include the relatively small sample size, unequal patient numbers across centers, and absence of individual follow-up data. Selection bias cannot be excluded, as more severely affected or more motivated patients may have been more likely to participate, particularly among those recruited via *Glandula*. However, given the small number recruited through this pathway, a major impact on the overall results is unlikely. To minimise selection bias, all consecutive eligible participants were offered study participation and underwent the same assessment and medical record review irrespective of recruitment pathway. Finally, the cross-sectional arm lacked a control group, as the prospective control cohort was too young to serve this function. However, this study arm intended to compare patients with and without recovery from adrenal insufficiency, which the design appropriately addressed.

In conclusion, this study adds to the increasing recognition of impaired occupational-health related aspects in patients with CS. As CS predominantly affects young women, and gender disparity continues to represent a persistent inequity in occupational and employment contexts, this population constitutes a particularly vulnerable patient group. Moreover, the discrepancy between documented functional impairment and the current German disability assessment practice, which generally regards postoperative patients as fully recovered, highlights a structural gap with direct consequences for social and occupational participation, underscoring the need to revise current assessment guidelines. Future large-scale longitudinal studies will be essential to determine factors influencing long-term socioeconomic outcomes in different healthcare settings. Individualised rehabilitation plans should be actively encouraged by treating physicians to ensure successful reintegration into professional and social life.

## Supplementary Information

Below is the link to the electronic supplementary material.

Supplementary Material 1

## Data Availability

The datasets generated and analysed during the current study are not publicly available but are available from the corresponding author on reasonable request.

## Declarations

### ORCID-ID

0000-0002-7323-9085.
