# Supplementary material for: Low work ability and high disability burden in Cushing’s syndrome: a multicenter cohort study
Source: J Endocrinol Invest. 2026 Jul 8;49(10):2721–34. doi: 10.1007/s40618-026-02966-5 (PMC13627271; doi:10.1007/s40618-026-02966-5)
Supplement: Supplementary file 1 [file 40618_2026_2966_MOESM1_ESM.docx]

**Supplement**

**Supplement 1a:** Current practice of German disability assessment guidelines (*Verordnung zur Durchführung des § 1 Abs. 1 und 3, des § 30 Abs. 1 und des § 35 Abs. 1 des Bundesversorgungsgesetzes (Versorgungsmedizin-Verordnung - VersMedV); "Versorgungsmedizin-Verordnung vom 10. Dezember 2008 (BGBl. I S. 2412), die zuletzt durch Artikel 1 der Verordnung vom 29. September 2025 (BGBl. 2025 I Nr. 228) geändert worden ist",* available online at: <https://www.gesetze-im-internet.de/versmedv/BJNR241200008.html?utm_>), original wording of the statute in German.

15.7 Chronische Nebennierenrindeninsuffizienz (Addison-Syndrom)

Sie ist gut behandelbar, so dass in der Regel dauernde Beeinträchtigungen nicht zu erwarten sind. Selten auftretende Funktionsstörungen sind analogen funktionellen Beeinträchtigungen (z. B. orthostatische Fehlregulation) entsprechend zu beurteilen.

Cushing-Syndrom

Der GdS wird bestimmt von der Muskelschwäche und den Auswirkungen an den verschiedenen Organsystemen (Hypertonie, Herzinsuffizienz, Diabetes mellitus, Osteoporose, psychische Veränderungen).

**Supplement 1b:** Current practice of German disability assessment guidelines according to German Health Care Act (*Bundesversorgungsgesetzes (Versorgungsmedizin-Verordnung - VersMedV)*), authors’ translation into English.

15.7 Chronic adrenocortical insufficiency (Addison’s disease)

It is well treatable, so permanent impairment is usually not expected. Rarely occurring functional disturbances are to be assessed in accordance with comparable functional impairments (e.g. orthostatic dysregulation).

Cushing’s syndrome

The degree of impairment (GdS) is determined by the extent of muscle weakness and by the effects on the various organ systems (hypertension, heart failure, diabetes mellitus, osteoporosis, and psychological changes).
